# The Foreign Journals: Surgery

**Published:** 1841-10

**Authors:** 


### SURGERY.

*Absence of the Nasal Duct, and its Artificial Formation.* By M. BÉRARD.

A MAN, twenty-one years of age, was admitted into the hospital Necker on account of congenital fistula lachrymalis. This fistula discharged a limpid transparent fluid, and caused continual epiphora. On pressing on the angle of the eye in the morning a muco-purulent liquid flowed from the fistulous orifice and from the puncta. The nostril of the same size was habitually dry; stimulating powders, such as snuff, becoming dry without exciting the secretion of the pituitary membrane. A stylet introduced into the fistulous orifice in the direction of the nasal duct would not pass, nor was it possible to penetrate its nasal orifice. No doubt could exist, therefore, of its congenital absence, and M. Bérard made an artificial nasal duct by piercing the os unguis after the manner of Woolhouse.

The inferior border of the internal portion of the tendon of the orbicularis being laid bare by incision, M. Bérard directed a trocar downwards, backwards, and inwards, perforating the internal wall of the orbit. The trocar was immediately replaced by a silver canula about half an inch long, enlarged at its two extremities, and on closing the mouth and nostrils of the patient, the air passed through the canula, showing that it was well placed. Three days after the operation the small wound had cicatrized; no bad symptoms followed. In two months, the patient having neglected the directions of the surgeon, returned with epiphora, when M. Bérard changed the canula, and in two months the epiphora had completely disappeared, and the patient was quite well in February last.

*Bulletin Général de Thérapeutique. Juillet* 15 *et* 30, 1841.

---

*New Artificial Leg.* Invented by M. LEPAGE, a Stonemason of Paris.

THIS artisan having had the right leg crushed by an enormous stone submitted to amputation, and being poor was obliged to follow another trade and became a shoemaker. The ordinary wooden leg without joint did not suit his new condition; he broke several, and he therefore contrived a leg with two joints, the one corresponding to the knee, the other to the hip. By means of a button applied to a spring, which descends laterally from the superior to the inferior articulation, these two joints are easily accessory to different movements, especially those of sitting down and rising up. The contrivance is very ingenious. The cost is about two guineas.

*Bulletin de l'Académie Royale de Médecine.* 15 *Juillet,* 1841.

*On the Curative Influence of Galvanism in some of the Organic Diseases of the Eye.*
By Dr. LERCHE, of St. Petersburg.

[THE following is the report made by Dr. Lerche, one of the principal physicians of St. Petersburg, on the application to opacities of different parts of the eye of the galvanic action which Dr. Crusell, a physician of Finland, was said to have employed with great success for the removal of other kinds of thickening, &c. consequent on inflammation.]

To Dr. Crusell of Finland is due the honour of having employed galvanism in a new and peculiar manner for the cure of organic diseases. Having been attracted by the treatise on the subject which he had sent to the Imperial Academy of Sciences, I had proposed to repeat the galvanic experiments instituted by him on the diseases of the lens in animals. The necessary apparatus was not yet completed when Dr. Crusell himself arrived at St. Petersburg; we therefore determined to experiment in his presence and with his assistance. For the subject of the first experiment we chose a complete leucoma of the cornea, which was deemed incurable, and in which, therefore, nothing could be lost, and perhaps something might be gained. The patient, a boatman sixty-eight years old, was in our hospital for chronic inflammation of the other eye. Our apparatus, according to the directions of Dr. Crusell, consisted of a simple galvanic circle of one zinc and one copper plate, both immersed in dilute sulphuric acid. The wire proceeding from the copper-plate, which we named the copper-pole, was placed in contact with the leucoma; that coming from the zinc-plate, the zinc-pole, was made to touch the patient's tongue, and the galvanic current was maintained for two minutes. The patient did not suffer in the least degree, neither did any unpleasant symptoms follow the operation; on the contrary, the chalky dulness at the margin of the cornea appeared somewhat thinner and clearer. The operation was repeated after three days, and we saw again a partial alteration in the state of the leucoma; and the patient, on his part, asserted that he had an increased perception of light.

The idea was now not remote to apply galvanism for the cure of opacities of the internal tissues of the eye. The results obtained by Dr. Crusell, in his experiments on the eyes of animals, strengthened our expectation of doing good, and we had opportunities enough of repeating them on eyes which had been regarded as almost or quite incurable. For safety's sake, however, we determined first to see the effects of the operation on some animal, and a young pig was chosen for the purpose. A fine cataract-needle fixed on the zinc-pole was passed through the cornea into the crystalline lens of the right eye, and the wire from the copper-pole was stuck into the ear. After thus remaining for four minutes the pupil began to grow turbid, and the operation was discontinued on the right but repeated on the left eye. Some days after we found completely-formed cataracts in both eyes, and the pig was quite blind.

Now, according to the theory, the artificially-produced dulness of the lens ought to be removed by a reversed galvanic action, and before we could feel justified in undertaking similar experiments in human eyes it was necessary to obtain the proof that such would be the case. After ten days, therefore, we proceeded to this second operation. In three minutes, (the galvanic current being maintained through the wires which were now so placed that the copper-pole was connected with the eye and the zinc with the ear), there appeared bubbles of gas in the pupil, and the process of solution seemed to be going on; the experiment was therefore at once brought to a close. The pupil appeared smoky and less dull. In four days it had almost regained its natural clearness; and the sight, so far as we could judge from the animal's action, was restored. In the cornea there was an opaque spot around the aperture made by the needle. This result at once determined us to make an experiment of a similar kind on the human eye.

We chose an old copper-smith from Finland, who had a long time previously been successfully operated on for cataract in the left eye. The right eye had presented a firm yellowish-brown capsulo-lenticular cataract, which had adhesions to the iris. It had been once depressed but it rose again; and in another attempt to break it up, nothing more could be done on account of its hardness, than the making of one vertical cut through its substance. This, however, rapidly closed, and for two months afterwards a variety of means were employed for the solution of the cataract without the least benefit. It was now very large, and lay close behind the widely-dilated, somewhat irregular, and immoveable pupil. The patient had a perception of light through it.

The galvanic operation was commenced in the presence of Dr. Crusell, in the forenoon of the 11th of November. It was very surprising to us to see, when the fine cataract-needle fixed on the copper-pole was inserted into the lens, and the zinc-pole laid on the patient's tongue, how, in less than a minute, the cataract swelled up, increased in size, seemed to press against the cornea, and then suddenly burst into three pieces, of which one was carried upwards and inwards, and another towards the temple, and the third projected downwards through the pupil into the anterior chamber. The triangular fissure left between them appeared clear and black. We now thought it best, as this was the first experiment of the kind on a living man, to stay the galvanic action. The patient could already see sufficiently to discern, with his right eye alone, both a finger held before it, and the face of a person standing in front of him. The operation lasted a minute, and neither produced pain or was followed by inflammation or any other bad symptoms.

Here the history ceases; the authors thought they had better leave the case with a limited amount of amendment, than run the risk of marring the advantage they had gained, and of throwing unnecessary discredit on the operation by going further. The facts, however, seemed to merit record as proofs that the plan is without danger, and offers some prospect of effecting a permanent cure of the disease.

*Medicinische Zeitung. Juni* 16, 1841.

---

*M. Ricord's New Urethro-plastic Operation.* By M. HELOT.

F. J. M. a shoemaker, twenty-six years old, came under the care of M. Ricord on the 16th of June, 1840. He said that when seven years old he took it into his head to tie up his penis with a thread, and after tying it tightly a little way in front of the scrotum, there followed on the next day a considerable swelling of the adjacent parts, in the midst of which the thread disappeared, cutting its way through the skin. To the swelling and division of the integuments, which cicatrized almost as fast as they were divided, there was added a retention of urine which lasted, the patient says, fourteen days. At the end of this time the urethra was divided by the thread, and a large quantity of urine came away; after this severe symptoms ensued, which, however, in six weeks entirely disappeared, leaving only a kind of constricted cicatrix around the whole circumference of the penis, with the two opposite ends of the divided urethra exposed. The patient was also affected with phymosis.

From this time, says the report, the patient's infirmity has scarcely troubled him. The emission of urine takes place in him as in those affected with hypospadias; during erections, which are complete, the penis remains perfectly straight, though the glans does not seem to participate in the erection so much as it should. Neither is this portion of the organ the seat of any of the sensations which are peculiar to it in its perfect state.

On the 1st of January this man was attacked by gonorrhœa eight days after connexion. The disease commenced in the posterior portion of the urethra, and it was not till four days afterwards that (the two orifices being in contact) the anterior part of the canal was affected. Then the discharge went on at once through the posterior aperture and both the anterior ones. When the patient came into the hospital the discharge was abundant and very purulent; the posterior or vesical portion of the urethra was the seat of pain in the passage of the urine; the other part was only a little painful on pressure.

The acute symptoms having yielded to reducing treatment, a daily dose of twenty-four grains of powdered cubebs was ordered. After a few days the discharge had considerably diminished in the posterior part of the urethra, but in the anterior it was unaffected; after six days' use of the cubebs it had entirely ceased in the former situation, but in the latter it still continued.

During this time the two portions of the urethra had been carefully kept from contact; but on now permitting them to come together the posterior part which had been cured was again infected by the anterior, and had again to be treated by cubebs. The anterior part was at the same time treated and cured by injections of nitrate of silver.

After this it was proposed to endeavour to cure the organic defect of his urethra by a plan somewhat similar to that which M. Segalas had once successfully employed. The patient was (on the 3d of November) placed as for lithotomy. A large catheter having been introduced by the opening of the posterior part of the canal into the bladder, an incision, commencing immediately behind the situation of the bulb, was made in the middle line of the perineum and into the membranous portion of the urethra, through which, with some difficulty, a female catheter was then passed into the bladder. This being fixed, M. Ricord next operated for the phymosis, that he might have more yielding integuments behind the glans; and then having revivified the edges of the apertures in the urethra he brought them together (after all bleeding had ceased) and fixed them by sutures. A bougie had, before drawing the sutures, been passed through the two parts of the urethra down to the catheter in the membranous portion.

After the operation the patient was kept very quietly, with his lower limbs somewhat raised, and in the evening he was bled. Next day the urine had flowed freely through the catheter in the perineum; the wound was wet with urine, and its edges were swollen; on the third day the sutures came out; there was no union, and the urine escaped by the fistulous opening as it did before the operation. The edges of the fistula, however, preserved a transverse direction, and such a thickness as to lead one to hope that fresh trials at union might be more successful. On the 15th of November a considerable abscess which had formed in the perineum was opened at the scrotum, and discharged for some time pus and urine; but after it had healed fresh attempts were made to induce all the urine to pass through the opening purposely made in the perineum by introducing into it each day a larger catheter. This method was at last successful, and on the 19th of January a new union of the aperture was made by twisted sutures over a bougie introduced to a shorter distance beyond the aperture than the first had been. Three days after this operation the two external pins were removed, and the union of the parts near them was found complete. Next day the two others were removed, and there remained but a very small aperture through which a little urine and pus flowed. This was touched with nitrate of silver, and in a week (on the 1st of February) it had closed; another then appeared which remained for some time, but was also ultimately closed.

On the 12th of February, three months and some days after the first operation the perineal catheter was withdrawn and a small one introduced through the whole urethra. Through this latter the urine now all passed, and the wound in the perineum rapidly healed. At the time of the report the patient was perfectly well; the penis had regained its form and discharged all its functions normally.

*Annales de la Chirurgie. Mai,* 1841.

---

*On the Application of Oil-varnish to the Immoveable Apparatus, in Fractures of the Limbs of Children.* By M. Tavignot, House-surgeon of the Hospital of Children's Diseases.

When the common immoveable apparatus is applied to the fracture of the lower limb of a young child, at the end of two or three days it becomes perfectly moveable by saturation with urine, and therefore totally incapable of maintaining the fractured parts in apposition. After some trials with glazed silk and spirit-varnish, which were soon permeated by the fluid, M. Tavignot found that oil-varnish answered the purpose extremely well. The best mode of using it is to apply the starch-bandage first, and this having become completely dry a layer of the varnish is to be smeared over its whole external surface, and also over its superior border and part of its internal surface by a fine brush. The limb is then to be exposed to the air in warm weather, and to artificial heat in winter. Another layer of varnish is to be applied in about two hours, being applied especially on the parts of the limb most exposed to the action of the urine. When the layer has dried the ordinary coverings of the limb may be replaced, and the urine then glides over the surface of the bandage with great facility without affecting it in the least. M. Tavignot has placed a bandage thus prepared in constant contact with urine, by putting it into a vessel full of this fluid without altering its consistence in any degree.

This is not the only advantage, for the apparatus can be removed without cutting the bandages, by placing the child in a warm bath, for the water insinuates itself between the bandage and the limb, and so softens the former by dissolving some of the starch, that the limb is readily unrolled.

Paper steeped in this varnish appears likely to prove highly useful to the surgeon in protecting parts exposed to the action of acrid or irritating fluids.

[The composition of this varnish is not given. It is probably the common size-varnish of our oil-shops.]

*L'Examinateur Médical. Août* 1, 1841.

---

*Successful Case of Excision of the Head of the Humerus, after Compound Fracture.*
By Dr. HELLO, of Cherbourg.

AN English boy, fifteen years of age, fell from a height on the 16th of April, 1841, and sustained a fracture of the humerus at the situation of its surgical neck. The lower fragment tore the integuments between the great pectoral and deltoid muscles, and appeared through the clothes. There was considerable hemorrhage at the moment of fracture, but the fracture was almost immediately reduced, and the bleeding ceased. On passing the finger into the wound it was found full of clots; the inferior fragment of the humerus was denuded of its periosteum to the extent of about six centimetres; the superior fragment, very short, offered a number of small splinters which were almost attached to the articular capsule. I at once determined to lay bare the shoulder-joint, with the double purpose of resecting the humerus and extracting the head of the bone. A semicircular incision was made, commencing a little below the coracoid process, following the line which separates the great pectoral and deltoid, turning backwards at the insertion of the latter which was cut to the bone, and remounting to the acromion along the external border of the deltoid. The flap thus formed was turned upwards, and the arteries nourishing it tied. The superior part of the humerus was then carried from the wound, (which was easily done by seizing the condyles to direct the bone,) the periosteum surrounded with the bistoury below the situation where it was torn, and after having placed a piece of pasteboard between the flesh and the bone, the denuded portion of the latter was removed by an ordinary saw. The posterior circumflex artery which bled freely was tied; and being cut so near the axillary trunk it is not improbable that this might render secondary hemorrhage frequent after this operation, the formation of the clot being prevented. The axillary artery was so superficial at the bottom of the wound that I should have passed the ligature around it, would not this have hazarded the success of the means employed to save the limb. The musculo-cutaneous nerve which had been torn was excised, the flap reapplied, three sutures inserted, and the parts were then kept in apposition by adhesive plaster, and covered with compresses of charpie, the whole being maintained by a bandage crossing under the opposite axilla. The forearm and hand were placed in a sling, and the arm was separated from the chest by a soft cushion of charpie. The patient went on without a bad symptom until the 19th, when there was some fever, with fetid and abundant suppuration. On the 23d there was less fever, and the suppuration more healthy. Two ligatures came away. Sutures removed. Internal part of wound cicatrized. All the ligatures had come away by the 29th. On the 1st of May a well-marked intermittent set in, for which quinine was given, and continued for several days. The wound had perfectly cicatrized by the 31st. The patient could seize small objects with the hand, and having recovered his health wished to return to England immediately.

This case is a very interesting one, showing that, under very favorable circumstances, a useful limb can be secured to the sufferer, and pointing out a very easy mode of performing the operation. The author states that he followed the same proceeding on a Turk at Marseilles, but he was sent to Smyrna on the fifth day, and the result has not been ascertained.

*Annales de la Chirurgie. Juillet* 1841.

---

### *On a new variety of Hernia: Hernia destitute of Peritoneal Sac.* By M. Dumeaux.

The following remarkable disposition of parts was observed in the body of a man between 55 and 60 years of age, who had a large scrotal hernia on the right side. After removing all the coverings of the tumour, the hernial sac, or what appeared to be the sac, instead of being a uniformly resisting membrane, presented very distinct muscular fasciculi, and the posterior wall of the cæcum was immediately recognized. On examination by the abdomen it was found that the cæcum instead of occupying the iliac fossa, was placed on the abdominal wall, and covered by peritoneum on one of its surfaces only. Its adherent wall was engaged in the inguinal canal forming part of the hernial tumour. The free wall, covered with peritoneum followed, being as it were invaginated in the former, and forming towards the abdomen a cavity or true hernial sac, in which seven or eight inches of intestine were engaged.

This singular species of hernia has never before been described. The preparation was presented to the Anatomical Society.

*Annales de la Chirurgie. Juillet,* 1841.

---

### *Two Cases of Strabismus cured without Operation.* By M. Beydler.

A woman, thirty-three years of age, regular in menstruation, had been affected with diplopia for six weeks. There was convergent strabismus of the right eye, apparently owing to paralysis of the external rectus. It manifested itself after noises in the head. Half a grain of strychnine was dropped into the eye daily, which was followed by amelioration; and complete reestablishment of the sight succeeded six applications of electricity, twenty or thirty sparks being disengaged at the external angle of the eye at each application.

A man, thirty years of age, after acute mental suffering caused by his trial in a court of justice, suddenly squinted with the left eye. He was acquitted, and after fifteen days the two eyes became again perfectly parallel.

*Annales de la Société de Médecine de Gand. Septembre,* 1840.

---

### *On the Radical Cure of Spina Bifida, by a New Operation.* By M. Dubourg, Physician to the Hospital of Marmande.

Having observed that the ordinary method of treating spina bifida was far from successful, and reflecting that when the soft parts over bones separated by arrest of development are brought together by suture, they have considerable effect in approximating the edges of bone, as in cleft palate, M. Dubourg concluded that by bringing together the soft parts over the defective spot in the spinal column, a radical cure might be effected by producing permanent approximation of the edges of the bony opening. The first case in which he put his ideas into execution proved unsuccessful, the child having died two days afterwards, but in two other cases the success was complete.

CASE I. In the spring of 1837 I was called to a female infant eight days old, who had a congenital pediculated tumour on the lumbar region, about the size of an apple. Its colour was livid from the development of a venous network, giving it the appearance of a vascular fungus. It was evident on examining the vertebræ that they were defectively formed, the edges of a bony opening being discovered. The tumour was opaque, its walls much thicker than usual, and it appeared that the arrest of development was confined to the last lumbar vertebræ, the child being otherwise healthy and well formed. Having the cautery in readiness in case of severe bleeding from the vascular coverings, and being prepared to prevent the sudden escape of the spinal fluid, an elliptical incision was made around the base of the tumour, when a quantity of reddish serum escaped, and the excision of the sac was readily effected. The finger passed readily into the spinal canal. The edges of the wound were brought together, and four needles being passed, the twisted suture was applied as in a case of hare-lip. The threads were twisted so as to exert as much traction as possible on the contiguous parts, and small compresses were placed at the extremities of the needles to protect the skin. The child cried sharply at the commencement of the operation, but as soon as the fluid escaped from the spinal canal it fell for a few minutes into a state of stupor. It cried again as the needles were applied, and by the time the dressing was completed it took the breast as though nothing had occurred. The needles and sutures were removed on the fourth day, when the edges of the wound were found to be united. Adhesive plaster was applied, and in fifteen days a strong cicatrix, forming a sort of solid button filling up the opening in the vertebræ, was all that remained of this reputed incurable disease. The examination of the removed tumour demonstrated that it was a cyst distended by fluid, communicating with the spinal marrow, bounded behind by the common integument and an expansion of the arachnoid and dura mater. The cavity was not in proportion with the volume of the tumour, for there were several layers of cellular and adipose tissue between its external and internal surfaces.

CASE II. The next case was similar, except that the swelling was situated over the junction of the last cervical and first dorsal vertebræ. The same operation was performed except that the sac was not perforated in the first incision, a lateral flap being formed with a narrow bistoury from within outwards, and the sac being then removed and the other flap formed by a second incision. The needles and sutures were applied as soon as possible to prevent the loss of any considerable quantity of the spinal fluid, and the access of air. The child recovered without an unfavorable symptom and continues well, eighteen months after operation.

The following are the conclusions drawn from these cases by the author, some of which, at least, are premature:

1. Some cases of spina bifida are susceptible of a radical cure.
2. Instead of abandoning to their fate most children born with this malformation, those should be selected in which this operation might be efficacious.
3. Although it is impossible absolutely to establish limits of incurability, every child born with a spina bifida, of which the opening of communication with the spine does not exceed an inch in diameter should be submitted to operation.
4. Ablation of the tumour and the twisted suture constitute the best operation.
5. It is well to open the spinal canal as late as possible in the operation, and to make the opening narrow.
6. When the sac is formed by the meninges, only the skin being atrophied over the tumour, the skin must be dissected on each side of the vertebræ, and the edges of the integuments united as in hare-lip.
7. The probability of reunion is proportionate to the size of the osseous opening and the general state of the patient.
8. The spinal canal may be opened, the spinal marrow laid bare, and a considerable portion of the fluid which bathes this important organ lost with impunity. *Gazette Médicale de Paris. Juillet* 31, 1841.

*Improved Method of Scleroticonyxis.* By Dr. Ruete, of Göttingen.

The first part of this operation is performed in the same way as in the ordinary proceeding for the depression of a cataract. But in the second part, the author does not endeavour, as most operators do, to pass the needle into the posterior chamber between the iris and the capsule (because this is neither a safe nor an easy proceeding), but he carries the needle, with its convexity turned forwards and its cutting edges upwards and downwards, about half a line from the uvea, and then when it has passed for a line or a line and a half into the eye, he makes with its cutting edge a vertical section through the capsule, and carries it on between the capsule and the lens till its point appears at the middle of the pupil. Then he turns the point of the needle forwards, strikes it through the anterior wall of the capsule, and cuts the latter from above downwards and from within outwards. The remaining stages are performed in the ordinary manner of depression, reclination, or breaking up.

The advantages of this method are stated to be as follows: The section through the lateral part of the capsule and the zonula Zinni prevents the tearing and stretching of those parts which in the ordinary method of operation is scarcely avoidable. There is no danger of injuring either the ciliary processes or the iris. The anterior wall of the capsule can easily be cut through, and there is no risk of capsular cataract remaining, for the four portions into which it is cut shrink up, and when the pupil is dilated with belladonna may be seen forming a grayish-white circle behind the ciliary body. All the injury inflicted being done by cutting, and none by tearing, it is very rarely that the operation is succeeded by inflammatory reaction.

The author has practised his new method successfully in twenty-eight cases.

*Holscher's Annalen.* Bd. iii. Hft. iv.

---

*Case of Pruritus Scroti cured by fresh Lemon Juice.* By Dr. Oppler, of Tarnourtz.

This was an extremely distressing case, that had resisted all internal and external means for ten weeks, depriving the patient of sleep, and producing incessant distress. The pruritus extended to the penis, and was accompanied by no primary rash, nor any perceptible local alteration except what was produced by the friction. A wash of diluted lemon-juice gave immediate relief, and after a few applications produced a perfect cure.

*Berlin Med. Zeitung. June* 30, 1841.

---

*On the Condition of the Nails in Fractures of the Limbs.* By Dr. Troschel.

Dr. Pitschaft mentions, in Hufeland's Journal, St. viii, the remarkable fact that the nails do not grow during the union of a fracture in any of the extremities, and that the commencement of their growth is a proof that the fracture has completely united. Dr. Lester is mentioned as the first observer of the fact. In the cases which have since occurred to me, I have been able to confirm its truth. In the first weeks after the injury one might be inclined to attribute the want of projection of the nails of the injured limb to the swelling of its fingers or toes; but this swelling not unfrequently continues for some time after the fracture is united, and then it may be seen that the nails again begin to grow, and that their having before projected less than those of the uninjured limb was actually the result of an arrest of their growth.

[We suspect that the defective growth has only an indirect connexion with the fracture. The growth of all the horny tissues bears a direct proportion to the degree of waste to which they are exposed; when a limb is fractured it is kept quiet, and its nails being guarded from the friction and other sources of waste to which they were before exposed, grow less rapidly than those on the other limb which are exposed as much or more than before. The subject, however, deserves notice.]

*Medicinische Zeitung. April* 28, 1841.

### *On the Cure of Ozæna.* By Dr. DETMOLD, of Hanover.

THE author says he has never failed to cure ordinary ozæna (by which he means the chronic coryza accompanied by a stinking discharge from the nose and a flabby relaxed state of the Schneiderian membrane) by the use of an injection composed of one or two drachms of chloride of lime rubbed up in a glass mortar with thirteen ounces of decoction of rhatany root, and strained off after standing for half an hour. About half an ounce of this must be injected into the nose three or four times a day with a syringe whose point is sufficiently long to carry the fluid high up into the nasal passages. The use of the remedy should be accompanied by the occasional administration of purgatives. It is very beneficial also in cases of chronic otitis with offensive discharge from the ear.

*Holscher's Annalen*, 1840. Bd. v. Hft. i.

---

### *On Hairs within the Eye.* By Dr. STILLING, of Cassel.

A blacksmith, a healthy man thirty-four years old, had the end of a wire driven with considerable force through the cornea and to the depth of half an inch into the interior of the right eye. Next day he had acute inflammation of the whole globe, and a high degree of fever, which were reduced by antiphlogistic remedies. Within a few days the wound in the cornea completely closed so as to leave but a small cicatrix; the chambers, which had been completely evacuated, were again filled, but a partial capsular cataract had formed. The sight, which had been entirely lost, improved considerably, and though at first it was double (apparently from the axis of vision being altered by an adhesion of the iris), it soon again became single and slowly increased in clearness.

About six weeks after the injury the attendant surgeon first discerned in the posterior chamber some hairs of different lengths, of which the author gives the following account from an examination made about four years after the accident: The left eye is perfectly healthy; in the right the iris is discoloured as if from a former attack of chronic inflammation. The pupil of the latter eye is irregular, and there is a partial capsular cataract. One hair commences a line below the horizontal diameter of the pupil and extends in an almost vertical direction (forming a slight arch, like that of the inner eyelashes) to the outer edge of the iris, where it comes upon the cornea. It is about five lines long and pointed at both ends; it is light yellow at its lower extremity and dark brown at the upper. The second hair begins near the vertical diameter of the pupil, on the lower and inner part of the pupillary margin of the iris, and extends obliquely upwards and outwards to the distance of a line and a half beyond the outer margin of the pupil. It is three lines long, and is pointed at one and probably at both ends; it is of a dark brown colour. A third hair lies just below that last described, and seems to come from the same root; it extends in the same direction, has a pointed extremity, is about three lines long, and of a yellowish-white colour. The appearance of two other hairs proceeds from some small black points attached to the capsule of the lens, and probably resulting from particles of uvea affixed to it and adhering with fine filaments of fibrin passing from it to the iris. The small cicatrix is still visible in the middle of the cornea, and is marked by a black point, which is probably the consequence of a small portion of uvea having been left in the wound when the wire was drawn out directly after the accident. The eyelashes and eyebrows are of the same colour as the hairs within the eye. The patient suffers no pain in the affected organ, and its sight is not more impaired than by the opacity of the capsule of the lens.

The author discusses the question whether these hairs were really produced within the eye as they sometimes are from growths on the conjunctiva, or whether they were carried into it by the wire, and had subsequently become adherent. He adopts the latter view, grounding his opinion chiefly on these facts: that there was no appearance of a growth from which they could originate; that they did not proceed from the same point; that they exactly resembled the man's eyelashes; that they did not grow; and that such a circumstance as the production of hair within the eye is not only unheard of but most improbable. [On the other hand, it is surely scarcely less improbable that a wire should carry eyelashes through the cornea and leave no cicatrix on the lid, and that these should become adherent, and yet not be remarked by a careful observer daily examining the eye for nearly six weeks.]

*Holscher's Annalen.* Bd. iv. Hft. iii.

---

*On the Employment of Nitrate of Silver in White Swellings.* By M Jobert, Surgeon to the Hospital St. Louis.

By a series of accurate and conclusive observations M. Jobert has shown that the best and most prompt means of overcoming articular pains in cases of white swelling, and to make the turgescence of the tissues disappear, consists in the external employment of an ointment of nitrate of silver. We have watched on fifteen patients the action of this remedy, in the wards of M. Jobert, and have been astonished at the prompt effects in long-continued and previously rebellious diseases. The treatment consisted in frictions on the diseased articulation with an ointment composed of thirty parts of lard to four of nitrate of silver. If the action of this be insufficient M. Jobert uses eight or twelve parts of the salt to thirty parts of lard. These ointments, designated by the numbers 1, 2, 3, constitute the whole of the treatment. Twelve or fifteen hours after the first employment of the ointment, and generally after the second friction, an eruption of small accuminated pustules appears, presenting a black point in their centre, and surrounded at their circumference by a small rosy areola. The liquid contained in the vesicle at first resembling thick milk, and rapidly assuming a yellowish-white appearance, afterwards becoming true pus. Each friction is accompanied by pains which last three or four hours. About the second or third day the skin becomes of a violet colour, and smarts acutely. The frictions must then be suspended, and not renewed until the parts are calmed. We do not enter into further details, as a full memoir on the subject is promised.

[It is very extraordinary that such accurate pathologists as the French should so generally continue to class as "*tumeurs blanches*" the very different diseases to which the joints are subject. We are thus left in doubt whether the above cases were scrofulous enlargement of the articular extremities of the bones, ulceration of cartilage, disease of synovial membrane, or of parts external to the joint.]

*Bulletin Général de Thérapeutique. Juin,* 1841.

---

*On Ecchymosis of the Eye and Eyelids as a means of Diagnosis in Injuries of the Head.* By Dr. Maslieurat Lagémard.

After the recital of several interesting cases the author arrives at the following conclusions:

1. That sanguineous effusion on the exterior of the cranium in the sub-aponeurotic cellular tissue, and before a line passed transversely from the posterior border of one external ear to the other, may appear on the exterior, determining ecchymosis in the eyelids, but not in the cellular tissue of the conjunctiva or of the globe of the eye.

2. That bodies which act directly on the eye, and which by their form are capable of compressing it from before backwards, may rupture the surrounding capillary vessels, and produce ecchymosis of the conjunctiva and eyelids.

3. When there is a fracture of the base of the cranium which is denoted externally by ecchymosis owing to extravasation of blood into the cavity of the orbit, this ecchymosis *appears first on the ocular conjunctiva,* afterwards reaching the eyelids in some cases but not in all.

*Archives Générales de Médecine. Juillet,* 1841.

### *On Dislocations of the Sternal Extremity of the Clavicle backwards.* By M. V. MOREL.

THE author has had three opportunities of examining this rare accident at La Charité and La Pitié, and from a comparison of these cases with the only three others published, has drawn up an essay, of which the following is an abstract:

The causes of this dislocation are forces acting *indirectly* by pushing the shoulders violently forwards, or by suddenly pulling the arm forward while the trunk is fixed, or *directly* by sudden blows on the front of the clavicle. The latter cause is by far the most rare.

The symptoms at the moment of the accident are extreme pain at the base of the neck, with syncope or a tendency to it, and generally more or less difficulty of breathing. The position of the limb is nearly the same as in fractures of the clavicle.

When the dislocated bone has passed backwards and downwards, its scapular extremity is always more than naturally prominent, and the clavicular end is lost sight of; but, if the patient be thin, and the parts not swollen, it can be felt pushed in behind the sternum. A depression more or less painful to the touch indicates the vacancy at the sternal fossa, and the external portion of the sterno-mastoid muscle is obviously turned backwards and inwards; but none of these last signs can be discerned if the patient be fat or if the injured parts be much swollen. The shoulder does not readily move, but if the whole strength of two men be applied to pull it backwards and outwards, the parts regain their natural relations, and the luxation is reduced; but the instant the extension is remitted, a rubbing sound and the reappearance of the deformity announce the reproduction of the displacement.

The dislocation backwards and downwards is very often by the efforts at reduction or other forces, converted into one backwards and upwards. In this case the end of the clavicle instead of being fixed is moveable, and, projecting inwards and upwards, forms above the sternum a small hard round tumour which moves with every motion of the shoulder, and may be easily reduced and pushed out of the joint again.

The reduction of the dislocation backwards and upwards then is perfectly easy; that of the dislocation backwards and downwards is best effected by pulling the upper part of the arm horizontally backwards and outwards while the elbow is held against the side of the chest. But the main difficulty is to keep the head of the bone, when reduced, in its place, and this may be best accomplished by M. Velpeau's bandage, which fixes the arm with its elbow on the front of the chest and the hand on the opposite shoulder, and is itself retained in place by a second bandage similarly applied and thoroughly starched. It must be worn for forty or fifty days.

*Annales de la Chirurgie. Juin,* 1841.

---

### *Case of Internal Intestinal Strangulation.* By M. GAUTRIC.

AT the sitting of the Anatomical Society, July 7, 1841, M. Gautric presented the pelvis of an old woman who had never had anything abnormal in connexion with the abdomen until some days before death, when she presented all the phenomena of internal strangulation. On examination it was found that the left ovary had contracted adhesions by its external extremity to the point where the peritoneum leaves the corresponding lateral surface of the bladder, thus forming a species of arch large enough to admit three fingers, and so disposed that the inclined plane of the internal iliac fossa leads directly towards it. A portion of small intestine about two feet in length was engaged under this arch and there strangulated. This adhesion was evidently produced by an old local peritonitis, for around the sigmoid flexure of the colon and between the uterus and bladder there were other filamentous adhesions. The fimbriated extremity of the fallopian tube was free.

*L'Examinateur Médicale. Juillet* 11, 1841.

### *Traumatic Pneumothorax, produced by violent Pressure on the Chest.*

Pneumothorax from disease of the lung and the production of pleuro-bronchial fistula is not uncommon, but it is very rarely produced by violence applied to the thoracic parietes. Hence the following case which lately occurred in the hospital of la Pitié is of some interest. A man, thirty-eight years of age, of robust constitution, was standing with his back to a post, between which and the wheel of a carriage in motion he was forcibly compressed, the wheel being applied to the anterior part of the chest. He fell senseless, and some hours after the accident presented the following symptoms: General prostration, intense dyspnœa, hard pulse, tympanitic resonance throughout the right side of the thorax, enlargement of that side with elevation of the intercostal spaces. A dry sibilant metallic râle occupied the middle part of the lung fixed at the same point, and recurring at each inspiration: scarcely any respiratory murmur; amphoric resonance towards the roots of the lungs. The whole external injury was ecchymosis with excoriation over the inferior angle of the right scapula. On the left side, where the pressure appears to have been less violent, the resonance of the chest and the nature of the respiratory murmur were not sensibly modified. There was considerable ecchymosis under the conjunctiva at the internal angle of the eyes. Metallic tinkling was very well marked on the next day. Seven bleedings were practised in five days from the time of the accident, the first to a pound, the second a little less, and on the four following days from half a pound to twelve ounces. Abstinence was rigorously enforced. The effusion was gradually absorbed and the patient is doing well.

The condition of the patient is explained by rupture of the lung and pleura pulmonalis, pneumothorax, inflammation of the pleura and formation of pus, with obstruction to the return of the blood from the head at the time of the accident; which would doubtless have produced rupture of the vessels of the pia mater and apoplexy, had the pressure been more violent, or continued for a longer time.

*Bulletin Général de Thérapeutique. Juin,* 1841.

---

### *Report on M. Louvrier's Treatment of Anchylosis by sudden and forcible Extension.* By MM. Thillage and Bérard.

The following is the substances of the above lengthy report. M. Louvrier's machine has been employed on twenty-two patients, of whom only three have experienced ill effects, all the others having escaped injury. Most of the patients suffered excessive pain at the moment of operation. In no case has the anchylosed articulation recovered entire freedom of motion. Those patients most successfully treated are obliged to use a staff in walking; one only walks without a stick, but the restraint is evident.

With regard to the unfortunate cases: in one female, in whom the anchylosis of the knee was complete and the limb fully flexed, the application of the machine was followed by a very considerable rupture of the skin, luxation of the leg upon the posterior part of the thigh, and abundant suppuration which terminated in death three weeks after the operation. At the necropsy the articular cavity was found full of pus, the popliteal artery intact, the popliteal vein full of pus and its coats thickened. Many muscles were ruptured and softened; the anterior crucial ligaments softened; one of the posterior softened, the other ruptured, attached by one extremity to the tibia and terminating at its free extremity by an osseous portion, which was evidently part of the condyle of the femur, fractured at the moment of operation.

Another patient suffered excessively acute pains at the moment of operation, and remained during some time in a sort of delirium occasioned by the suffering. Gangrene commenced on the next day, but was limited by the efforts of nature alone, and the patient is actually cured.

In a third case, that of a young woman in whom the anchylosed limb was fixed at a right angle, the straightening was incomplete. M. Louvrier applied a piece of wood to the anterior part of the knee by means of which he hoped to press the limb into its natural position; but an eschar formed on the next day and the patient died in six weeks.

In another patient who died from other causes, the articular extremity of the tibia was found to be luxated upon the posterior part of the femur, the internal condyle of the latter being fractured.

The number of these accidents, however, being small [!], the opinion of the reporters would be less unfavorable, were the ill effects balanced by real advantages; but as the limb after operation is as immoveable as an artificial support, they conclude:

1. That the application of the machine of M. Louvrier is followed by an instantaneous straightening (*redressement*) of the anchylosed limb.

2. That it is not ordinarily followed by any severe symptom, primary or consecutive.

3. That when these accidents are produced they are frightfully severe, and are ordinarily followed by death.

4. That none of the patients operated upon by this method have entirely recovered the free motions of the anchylosed articulation. We therefore report to the minister that the machine of M. Louvrier, although ingenious, is dangerous in application, for it will be always impossible to determine the nature of the anchylosis and to foresee the conditions which would offer some chances of success for its employment.

[The barbarousness of this most heroic chirurgery needs no comment from us to prevent its adoption. The facts, however, are well worthy of record.]

*Bull. de l'Acad. Roy. de Méd.* Nos. xiii-xiv-xv-xvi. *Avril* et *Mai*, 1841.

---

*On the Action of the Muscles of the Eye, and the cure of Myopia by division of these Muscles.* By MM. PRAVAZ, BONNET, GUERIN, &c.

I. The division of the great oblique in certain cases of strabismus having diminished the myopia which ordinarily accompanied this deformity, M. Phillips has been led to conclude that this muscle elongated the antero-posterior diameter of the eye, an elongation necessary for distinct vision at a short distance. M. Bonnet, who has cured several cases of myopia by dividing the lesser oblique, has assigned to the latter the same function. M. Pravaz assures us that they can have no such action, and proceeds to state that hitherto it has been supposed that myopia was owing to one of three causes and perhaps to the three united; an increased convexity of the cornea, an active or passive depression of the crystalline lens (according to its transverse *limbe*), or too great a distance existing between the lens and retina. M. Guerin, however, supposes that in vision at a short distance the eye is flattened from behind to before by the action of the recti muscles, while M. Pravaz concludes that the power of both recti and oblique, with the elasticity of the envelopes of the eye, perfectly accounts for all the phenomena of the appropriation of the refractive surfaces of this organ to variations of distance, and reconciles in theory the two methods which have been employed in the cure of myopia. The simultaneous contraction of the recti compresses the eye laterally, and also draws it from before backwards, the latter action being much the more powerful, but antagonized by the contractions of the oblique which thus indirectly tend to elongate the globe. The supposition that the recti cause myopia by diminishing the antero-posterior diameter of the eye is contrary to all optical principles, and its fallacy is shown by the success of the operations of Phillips and Bonnet; for according to this hypothesis the section of the oblique muscles would augment the myopia by removing one source of resistance opposed to the action of the recti.

*Archives Générales de Médecine.* *Mai,* 1841.

II. The case of M. Bonnet alluded to in the above paper was related at the sitting of the Academy of Sciences on the 29th of March last. He only performed the operation once, and with very little success. The case was that of a young man who had been short-sighted for two years, and suffered from some symptoms of amaurosis, and the only good effect of the operation was that the patient did not see sparks before his eyes as he had done before.

III. The following letter (abridged) on the cause and surgical treatment of myopia was addressed to the Academy of Sciences by M. Guerin, March 15, 1841: 1. There exist two species of myopia, the mechanical or muscular, and the optic or ocular. The mechanical results from the primitive shortness or active retraction of the muscles of the eye. 2. In the mechanical myopia the shortened muscles are the four recti together or two or three only. 3. When only one rectus is feebly contracted, or two or more irregularly contracted, strabismus coexists with myopia. 4. The characters of mechanical myopia are furnished by the form and movements of the eye. The anterior half of the globe is conical, the cornea representing a segment of a sphere the radius of which is much smaller than the segment of the eye which it fills up. The lateral parts of the globe are depressed, flattened in the situations of the shortened muscles. The movements of the two eyes are more or less limited upwards, downwards, inwards, or outwards, according to the number of muscles affected and their degree of shortening. 5. The treatment of mechanical myopia consists in the subconjunctival section of the short or retracted muscles. I have practised this operation several times in cases of simple myopia, and in myopia complicated with strabismus. Among the most remarkable cases I will cite that of a man fifty years of age, affected with slight divergent strabismus. He could read with No. 3 glasses; three days after the operation he could read readily without glasses the characters of the *Moniteur.* In another case a young man, aged eighteen, whose mother and grandmother had been shortsighted, before operation could not distinguish Roman characters at the distance of about five inches, but he read them readily at a greater distance with glasses No. 7. Three days after the division of the two internal and external recti he began to read without glasses, the same characters at the same distance, and could distinguish at the distance of ten yards objects which, before the operation, he could not see at all. On the ninth day from the operation the patient could read without glasses roman characters at the distance of eighteen inches, and large roman capitals at the distance of a yard, but they appeared smaller to him than before the operation. He distinguishes pretty clearly at the distance of 100 yards large objects, as a dog or a statue, although he cannot see the same objects with glasses No. 7, and but very confusedly with No. 13. The eyes as yet do not accommodate the focus to all intermediate distances, and this circumstance coincides with a reunion and contraction still incomplete of the divided muscles. M. Guérin then goes on to expound the theory we have before alluded to.

*Journal des Connaissances Médico-Chirurgicales. Mai,* 1841.

[M. Cuvier has practised with success the operation proposed by M. Guérin in four cases, and in one in which the patient offered signs of contraction of the inferior oblique, this muscle was divided. (*Annales d'Oculistique, Avril,* 1841.) M. Bonnet has performed his operation in three other cases, and although in two there was some improvement, in none was the cure complete. We believe, however, that our countryman, Mr. Adams, claims priority in applying myotomy to the cure of myopia, having performed his first operation in a short-sighted patient in August, 1840. See a notice of his book in the present Number.]

---

*Results of Operations for Stammering in France.* By MM. Velpeau and Bonnet.

The following extracts will confirm the justice of the observations made in our last Volume on the lately-introduced and lately-discarded operations for stammering. At the date of M. Velpeau's paper (June 1st), nearly 200 patients had been operated on in France; the modes of operating were already ten in number; the beneficial results obtained by others were even less than in M. Velpeau's patients.

I. *Experience of M. Velpeau.* The first patient cut was in three months in the same state as before the operation. In the second there was but very little amendment. The third and fourth remain cured (at the date of his paper). The fifth, who was at first much benefited, now stutters again very much. The sixth is improved but still stammers. The seventh, in whom the dorsum of the tongue was tied, and who was for some time benefited, now stammers as much as ever. The eighth, in whom the arches of the palate were cut, is in the same plight. The ninth, a physician, whose genio-glossi were divided, was in three weeks as bad as ever. A tenth after remaining well for twelve days, returned in a fortnight again stammering. The eleventh and twelfth gained but a very little advantage by the operation. The thirteenth had severe hemorrhage and then vast abscesses in the neck, but still was not relieved of his infirmity. Four others in whom the anterior edges of the genio-glossi were divided, and in whom the stammering previous to the operation was not very bad, considered themselves completely cured, and in fact did speak much better than before.

The dangers of the operation are hemorrhage and subsequent acute inflammation. [Both have occurred with sufficient frequency and severity to render it unjustifiable to proceed to an operation which does not afford a good prospect of benefit to the patient. From the reports in other journals we learn that not less than three patients have died of one or other of these accidents.]

From his own observations M. Velpeau concludes that the excision of a V from the tongue offers but a slight chance of remedying the stammering; that the destruction of a transverse portion by ligature does not succeed better; that the section of the arches of the palate, or of the glosso-pharyngei muscles is equally unavailing; that the prolonged division of the frænum often succeeds, without exposing the patient to any real danger, in rendering the speech more easy and in partly removing certain varieties of stammering; and that the section of the genio-glossi near their attachment to the jaw is sometimes almost perfectly successful, but is often ineffectual, and often of only partial utility.

*Annales de la Chirurgie. Juin,* 1841.

II. *Experience of M. Bonnet.* Of forty-two patients in whom the genio-glossi muscles have been divided by the subcutaneous method, two had the speech so confused that it was difficult to understand what they said; in these no effect resulted. The same want of success followed in four cases where the impediment to speech resulted from defective inspirations or expirations; the patients did not repeat the same syllables, but they were immediately arrested at the commencement or the middle of the words, the air not passing to or from the chest. In such cases we can hope for or obtain nothing from division of the genio-glossi.

Thirty-six of the operations were on true stammerers, that is to say persons who repeat the same syllable several times. Of this number six were more than thirty-two years of age, and the results were completely negative. We therefore conclude that we should not operate on those who have confused speech, nor on those where impeded respiration is the cause of the difficulty of speaking, nor on true stammerers who have exceeded thirty-one years of age.

Of thirty stammerers under thirty-one years ten were completely cured, eleven extremely ameliorated, and seven experienced no advantageous result.

Of the ten who were cured one had returned to his former state from twelve to fifteen days after the operation. Another who stammered excessively, and whose cure was as it were miraculous, found his defect reappear very gradually towards the fifth week, but two months after the operation he may still be placed among those greatly relieved though not among those cured. The others continue perfectly well.

Of the eleven greatly relieved, two relapsed, but were improved after a time. The others also gradually improved.

*Bulletin Général de Thérapeutique. Juillet* 15 *et* 30, 1841.

*Application of the Subcutaneous Method to the Operation for Strangulated Hernia.*
By M. Jules Guerin.

In this case the hernia was a congenital epiplocele which had been strangulated for three days. The usual means of reduction had been applied, and the tumour had become hard, engorged, and the seat of commencing inflammatory action. After division of the two rings and of the antero-superior wall of the inguinal canal, the reduction was immediately effected. The wound did not inflame, nor did the slightest febrile symptom follow. The patient was able to rise on the eighth day, taking care to wear a bandage.

*Gazette Médicale de Paris.* 7 *Août*, 1841.

---

*Ligature of the Temporal and Facial Arteries in a Case of Epilepsy.*
By M. Velpeau.

A man, thirty-six years of age, who had been affected with epilepsy for seven years, which followed a fright, was admitted on the 29th of March last into the hospital of *La Charité*, under the care of M. Velpeau. The attacks had occurred eight or ten times in a month, but daily for the last three months. He had some very severe attacks in the hospital. On the third day from his admission, M. Velpeau, emboldened by some facts scattered here and there in science, and which have hitherto passed almost unnoticed, tied the two temporal arteries. On the same day the patient had another fit, but slight, and on the following day he was perfectly tranquil. On the 4th of April M. Velpeau compressed the two facial arteries on the borders of the inferior maxilla. The fit did not return, and on the 5th of April the surgeon tied the two facial arteries. The patient quitted the hospital on the 15th of April, only having had one fit since the 5th, although for months he had not passed a day without at least one attack. He has promised to return to the hospital occasionally. The case is interesting, though further observation is of course necessary to show whether the ligature of the arteries was the cause of the cessation of the fits.

*Bulletin Général de Thérapeutique. Avril* 15 *et* 30, 1841.

---

*Good Effects of the Extract of Belladonna in the Reduction of Paraphimosis.*
By Dr. Mignot, of Bordeaux.

A child, three years and a half old, was the subject of severe paraphimosis; the glans red, swollen, and tender; the prepuce strongly drawn back, forming a thick and apparently adherent ring, the constriction of which completely stopped the circulation. This state had lasted eight days, and the sufferings were excessive. Reduction being impossible, leeches were applied to the perineum and hypogastrium; cooling drinks, emollient enemata, cataplasms, lotions, and hip-baths were used, but they only gave slight relief and but for a short time. The strangulation became more menacing, and all the symptoms were aggravated; the glans was bluish and gangrene was threatened, when M. Mignot employed frictions around the glans, with an ointment composed of thirty parts of simple cerate to twelve parts of extract of belladonna. Under the influence of this remedy the circle of constriction relaxed, dilated, and the tissues gradually recovered their normal condition, without loss of substance or suppuration following.

The second patient had acute balanitis, brought on by a severe gonorrhœa, and followed by paraphimosis. The patient refused operation although gangrene was threatened, when the belladonna was applied, which induced relaxation and rapid amendment. It was also applied in a case of phimosis accompanied by chancres and a sympathetic bubo, and three days after its employment the dilatation of the preputial orifice was complete.

*Bulletin Général de Thérapeutique. Avril* 15 *et* 30, 1841.

*On the Development of Cryptogamia in the Tissues of living Vertebrata.*
By MM. ROUSSEAU and SERRURIER.

IN a memoir on the Diseases of the Organs of Voice by the above gentlemen, it is related that a male paroquet affected with laryngeal and pulmonary phthisis died in 1834, and on examination there was found in the abdomen between the intestines and vertebral column a species of false membrane, on which there was a greenish and pulverulent mouldiness, which adhered so slightly that on blowing it disappeared as the finest and lightest powder. This mouldiness has been several times observed in different parts of the body; most frequently in the pelvis, between the kidneys and the viscera, on the principal vessels of the heart, between the ribs and the lungs. Pigeons and fowls are most particularly affected, especially those inhabiting cold and moist places, and in rainy seasons. Nevertheless this phenomenon has occurred in animals of a different organization, in a hind (*cervus axis*) and in the earth tortoise, a native of the Indies (*testudo indica.*) A similar fact has recently been communicated to the academy by M. Deslongchamps.

At the sitting of the Royal Academy of Sciences, July 5, 1841, M. Dumas stated that Dr. Gruby had discovered that one of the varieties of porrigo is owing to the development of a vegetable beneath the epidermis. M. Breschet remarked that the *porrigo favosa* was the form studied by M. Gruby.

*Gazette Médicale. Juillet* 17, 1841.

M. Kettner has since written to the academy to claim for Dr. Schönlein, successor to Hufeland at Berlin, the priority of indicating the existence of a vegetable in the crusts of *porrigo favosa*, a fact which was published by him in *Müller's Archiv*. M. Kettner states that Dr. Schönlein has established in many cases of *porrigo lupinosa* the vegetable nature of this affection, and therefore the discovery cannot be attributed to M. Gruby.

*Gazette Médicale. Juillet* 24, 1841.

[M. Gruby has made very minute observations on the physical and chemical properties of this vegetable, and has succeeded in inoculating it on cryptogamia, thus transmitting disease from man to vegetables, but only succeeded once in seventy-six trials. He failed to communicate the disease by inserting the vegetable into the arm. This subject is so interesting and important in connexion with the subject of animate contagion, that we shall wait with anxiety until the Report of the committee is published.]

---

*On Contraction of the Vesical Orifice of the Urethra.* By M. CIVIALE.

THE object of the author, in a very long paper, is to describe a particular condition of the vesical orifice of the urethra, frequently met with, especially in old men, resulting sometimes from morbid states of the prostate; sometimes from a sort of semilunar valvular fold of mucous membrane, elevated from the inferior surface of the neck of the bladder, and constituting a barrier which opposes the flow of urine or the introduction of instruments. This valve presents different degrees of thickness and elevation, sometimes being eight, nine, or twelve lines in height. It appears to result from elevation of the mucous membrane in consequence of prostatic engorgements. The treatment consists in dividing the barrier, from its free border to the base, or puncturing the base and cutting to the free border. M. Civiale has constructed instruments for this purpose resembling those used by Mr. Stafford for the division of stricture.

*Bulletin Général de Thérapeutique. Mai* 15 *et* 30, 1841.

